# The Impact of Climate Change on Forest Development: A Sustainable Approach to Management Models Applied to Mediterranean-Type Climate Regions

**DOI:** 10.3390/plants11010069

**Published:** 2021-12-27

**Authors:** Leonel J. R. Nunes, Catarina I. R. Meireles, Carlos J. Pinto Gomes, Nuno M. C. Almeida Ribeiro

**Affiliations:** 1 PROMETHEUS—Unidade de Investigação em Materiais, Energia e Ambiente para a Sustentabilidade, Escola Superior Agrária, Instituto Politécnico de Viana do Castelo, Rua da Escola Industrial e Comercial de Nun’Alvares, 4900-347 Viana do Castelo, Portugal; 2 MED—Mediterranean Institute for Agriculture, Environment and Development, Pólo da Mitra, Universidade de Évora, 7006-554 Evora, Portugal; cmeireles@uevora.pt (C.I.R.M.); cpgomes@uevora.pt (C.J.P.G.); 3 Departamento da Paisagem, Ambiente e Ordenamento, Universidade de Évora, 7000-671 Evora, Portugal; 4 ICT—Instituto de Ciências da Terra, Universidade de Évora, Rua Romão Ramalho, 59, 7002-554 Evora, Portugal; nmcar@uevora.pt; 5 Departamento de Fitotecnia, Universidade de Évora, 7000-083 Evora, Portugal

**Keywords:** climate change, forest ecosystems, sustainable forest management, Mediterranean forests

## Abstract

Forest ecosystems are divided into three major groups: boreal, temperate, and tropical. These can be subdivided according to the particularities of each type due to its relative location (littoral, mountain, etc.), climatic conditions, or even geological substrate. Climate change affects each type of forest ecosystem differently. However, it seems to affect temperate forests in Mediterranean-type climate regions more intensely. These regions are located over several continents, with major impacts of increased temperature during summer and decreased precipitation during winter. This situation affects Mediterranean forest ecosystems by increasing the risk of fires, which arise more frequently and are more severe. In addition, the emergence of pests and the spread of invasive species are well-known problems affecting these ecosystems. All of these conditions contribute to losses of productivity and biodiversity. To avoid the destruction of forest resources, and since Mediterranean-type climate regions are considered climate change hot spots with increased vulnerability to disturbances, the implementation of adaptive forest management models could contribute to increasing the resilience of such forests, which could also contribute to mitigating climate change.

## 1. Introduction

**Figure 1:**
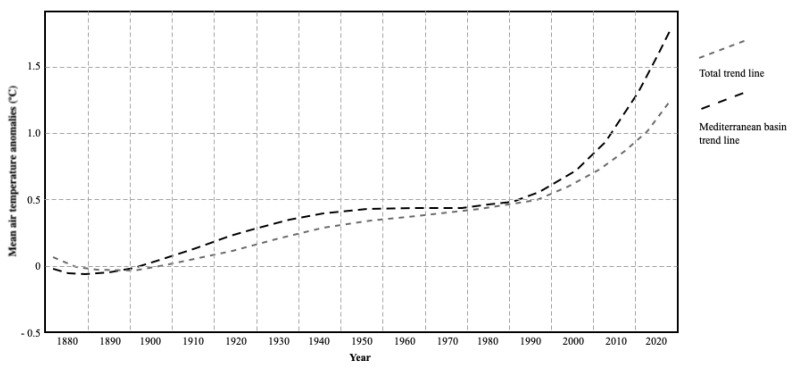
Temporal distribution of annual mean air temperature anomalies on the period 1880–1899 [6].

Climate change is considered the most significant challenge humanity has ever faced [1]. This perspective attaches great importance to the matter, leading to the launch of several global initiatives, with the intention to find solutions to mitigate the problem, specifically through paths that minimize the consequences [2]. The most visible and reported effect is undoubtedly the increasingly recurrent frequency with which extreme weather events occur, directly affecting the daily lives of people all over the world [3]. Example of this include the change in monsoon cycles, which subsequently changed agricultural cycles in Southeast Asia, significantly impacting food production, and prolonged drought periods in Mediterranean-type climate regions, with a direct impact on the increased risk of rural fires, as shown by recent events in California (USA), Portugal, Spain, and Greece, caused by the increasing air temperature in the Mediterranean-type climate regions, as shown in Figure 1 [4,5].

Forests are of particular importance as they are some of the most important ecosystems on the planet and are the habitats for an endless number of species. Table 1 shows the distribution of different types of forest ecosystems distributed by the respective climatic domains.

However, as this is a global-scale problem, with the capacity to change all biological, geological, physical, and chemical cycles of the entire Earth system, the impact on forests is also a reality, acting on these systems in a distinct but progressively way, altering the way forests evolve [7]. This evolution, as reported in several studies, presents a profound change in climatic parameters on a regional scale, causing changes in ecosystems and leading to, for example, the replacement of native species by exotic species, which, coming from regions presenting edaphoclimatic conditions closer to those observed now, may have higher competitive capacity, thus replacing native species in the ecosystem [8]. This leads to the loss of biodiversity, but it also causes damage to populations [9]. Such damage can be caused directly, for example, by the loss of productivity of a particular forest species, but also indirectly, by increased recurrence of rural fires, which in turn can accelerate the transformation of forest cover toward the development of pyrophytic species [10]. This type of replacement contributes even more to changing the type of forest, leading to the destruction of native forests and enhancing their replacement by exotic invasive species [11].

The adaptation of forests to new constraints because of climate change not only has an impact on aspects related to the stability of ecosystems, but also has a direct impact on populations living in and on the periphery of forest spaces. These alterations act as disturbances to ecosystems, such as the frequent occurrence of extreme heat events, which enhance the aridity of soils, and changes in rainfall, which make agricultural livelihoods more precarious, as indicated by Serdeczny et al. [12]. As those authors also indicate, the precariousness of rural systems can, in turn, lead to the growth of migratory movements originating in rural areas toward urban centers, leading to the growth of urban areas, with subsequent changes in land use. Contrary to what can be expected, forests located at higher latitudes may benefit from increased atmospheric carbon dioxide concentrations and rising temperatures, with expected significant increases in growth and wood production rates, at least in the short to medium term, as indicated by Lindner et al. [10]. On the other hand, increasingly frequent disturbances in the remaining forest regions of the planet will cause adverse effects that will be felt severely by people, with subsequent social impacts.

Thus, in the first part, this paper is aimed at analyzing the identified impacts of climate change on forests, namely those related to forest fires and invasive species dispersal, and in the second part, pays particular attention to forests located in regions with a Mediterranean-type climate. It is also aimed at understanding the impact of such evolution on the ecosystem services that these forests provide. Finally, it analyzes how forest management models can contribute to the sustainability of forest resources and which models can be more beneficial to counterbalance the impacts of climate change in forests in Mediterranean-type climate regions.

## 2. Forests in Mediterranean-Type Climate Regions

### 2.1. Mediterranean-Type Climate

The Mediterranean climate, or the Mediterranean-type climate when referring to regions not located in the Mediterranean basin, is characterized by rainy winters and dry summers. However, the apparent regularity has undergone visible changes in recent years associated with climate change, causing a reduction in precipitation during the winter, as mentioned by Tuel and Eltahir [13]. According to those authors, these regions stand out because of the magnitude and significance of the winter precipitation decline, with the reduction reaching up to 40% in some regions. This loss of precipitation could place profound limitations on water resources, which will constrain the capacity of regions to produce food, thus affecting populations that are often lacking in water, creating social instability. This recent acceleration of climate change accentuates the already frequent and profound environmental problems in Mediterranean-type climate regions caused by the combination of changes in land use, pollution, and loss of biodiversity [14]. Cramer et al. point out that in the coming decades, there will be a scenario of increased risk in strategic and vital areas with regard to the availability of water, preservation of ecosystems, food production, and population health and safety [6]. Due to their assumed importance, these questions require careful analysis, allowing the acquisition of in-depth knowledge about the climate and how it will evolve in each Mediterranean-type climate region.

Several studies have characterized the various changes observed in recent years in these regions. For example, Lionello and Scarascia [15] indicate that global temperatures have warmed, and decadal variability leads to significant uncertainty, preventing the identification of long-term links between precipitation and global temperature. On the other hand, Tramblay et al. [16], based on assumptions raised by other authors, developed a model to better understand the impact of global changes over a century. These changes necessitate serious consideration of the impact of climate change in different areas, particularly those that directly affect people’s lives. Even though it is a type of climate with similar characteristics in all regions, the geographic distribution of these regions over different continents necessarily implies specificities inherent to each one, and even within regions, some differences must be taken into account. In this way, for the construction of forecasting models, the collection of temporal data series is assumed to be a handy tool on which climate evolution models can be built, which can later be used to predict the evolution of concrete situations, such as forest ecosystems. The adaptability of different species to climate change has been the subject of in-depth studies, such as the work developed by Klausmeyer and Shaw [17], in which they analyze the potential for species in Mediterranean ecosystems worldwide to adapt to climate change, with an emphasis on habitat loss.

### 2.2. Mediterranean Forests

Mediterranean-type climate regions naturally present high biodiversity, which has attracted much attention in recent years, especially in terms of studying the effects of climate change. This biodiversity is the result of differential speciation and extinction rates during the Quaternary, which was influenced more by the incidence of fires and the severity of climate change than by environmental heterogeneity, according to Cowling et al. [18]. The authors also draw attention to the fact that all Mediterranean-type climate regions have many rare and locally endemic taxa where small populations survive, many of which are threatened by habitat transformation. This diversity seems to contribute to greater productivity of forests, as pointed out by Vilà et al. [19]. However, according to the same authors, functional group richness has increased wood production only in sclerophyllous forests. However, despite all of the biodiversity identified in these forests and the recognized high productivity, Mediterranean forests are very fragile and vulnerable to numerous threats such as fires, over-exploitation, deforestation, and degradation, which, according to Pahali et al., are currently being accentuated in the context of climate and land-use changes [20]. The permanent search for resources provided by Mediterranean forests serves to increase the value attributed to these resources, although, as pointed out by Croitoru [21], most of them are poorly recognized.

**Figure 2:**
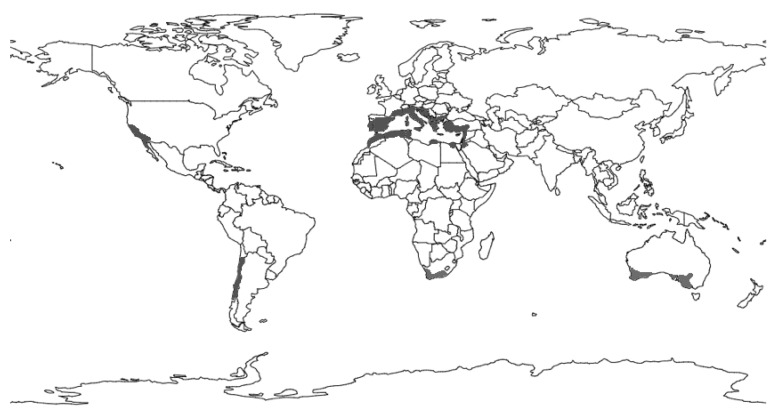
Location of the Mediterranean-type climate regions, which are distributed by Central Chile, California and Northern Baja California, Southwest and South Australia, Western Cape, South Africa, and the Mediterranean basin.

Currently, there are Mediterranean forests in five regions spread over several continents: in the Mediterranean basin, with forests distributed across various countries in Europe, Africa, and Asia; in California, North America; in Chile, South America; in South Africa, Africa; and in Australia, Oceania (Figure 2). Despite the broad geographic dispersion, all of these regions share a set of similarities, with increased aridity, mainly by decreased precipitation but also by higher temperatures, as the main threat to the diversity and survival of Mediterranean forests, as pointed out by Peñuelas et al. [22]. This weakness was previously recognized by several authors, such as Scarascia-Mugnozza et al. [14], who recommend the implementation of careful strategies for conservation and management. As those authors mentioned, this approach implies the need to identify appropriate silvicultural and management strategies, which will have significant effects on defining the criteria for sustainability and eco-certification. Issues of a social nature must also be considered as determining factors for effective forest conservation. Otherwise, it will be impossible to control forest fires and landscape degradation. Baeza et al. [23] highlight human action as the primary disturbance in the origin of fires and changes in land use, which play a critical role in the current state of vegetation. For example, fires create vegetation patches in different successional states, whereas land use and soil type define different shrubland types in terms of their specific composition [24].

### 2.3. Main Climate Change Disturbances in Mediterranean-Type Climate Regions

#### 2.3.1. Framework

**Figure 3:**
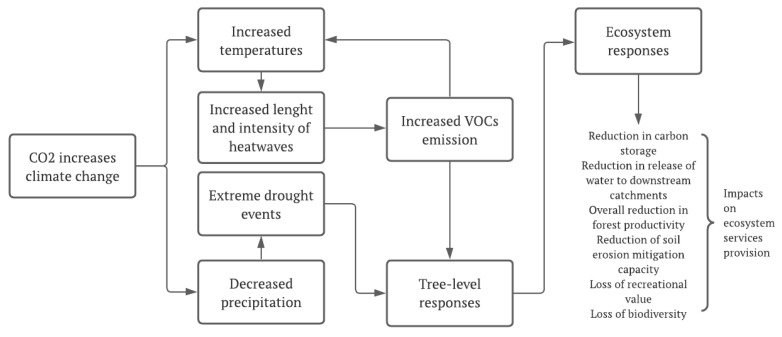
Tree- and ecosystem-level responses in Mediterranean forests to environmental changes associated with climate change and their impacts on ecosystem service (ES) provision [27].

Despite climate change being a global phenomenon, the consequences of its manifesting all over the planet, mainly in the form of extreme weather events, are felt more intensely and severely in some specific regions. These consequences will generate a set of responses at different levels, as presented in Figure 3. Giorgi [25] called these regions climate change hot spots. The author developed the so-called Regional Climate Change Index (RCCI) based on regional mean precipitation change, mean surface air temperature change, and precipitation and interannual variability change. Using this index, a set of regions were identified as hot spots. Among all analyzed regions, the Mediterranean and northeastern Europe emerged as the primary hot spots, followed by high-latitude regions in the Northern Hemisphere, along with Central America, the most prominent tropical hot spot. Southern Equatorial Africa and the Sahara are the major African hot spots, and eastern North America is the most important hot spot on that continent. These projections were later confirmed by other authors, such as Georgi and Lionello [26], who again identified a robust and consistent picture of climate change over the Mediterranean, consisting of a pronounced decrease in precipitation, especially in the warmer season.

Diffenbaugh et al. [28] looked beyond this evidence and analyzed the reasons for the intensification of climate change in Mediterranean regions, and concluded that higher greenhouse gas concentrations increase heat stress risk in these regions, with the occurrence of extreme heat increasing by 200% to 500%. The authors pointed out the preferential warming of the hot tail of the daily temperature distribution as the cause of intensified heat stress, with the 95th percentile maximum and minimum temperature magnitude increasing more than the 75th percentile magnitude. Following this work, Diffenbaugh and Giorgi [29] modelled different scenarios with varying temperature limits for different levels of global warming, and found that the Mediterranean region emerged as a prominent regional climate change hot spot in response to intermediate and high levels of forcing. This knowledge of possible scenarios can help to inform mitigation and adaptation decisions by quantifying the rate, magnitude, and causes of the aggregate climate response in different parts of the world.

It seems that the Mediterranean regions will undergo intense changes in their climate patterns, with several studies pointing in this direction. Lionello and Scarascia [15] analyzed the relationship between climate change in Mediterranean regions and global warming, and noted that during the 20th century, (i) Mediterranean and global temperatures warmed at a similar rate until the 1980s, and (ii) decadal variability led to large uncertainty preventing identification of long-term links between precipitation in the Mediterranean region and global temperature. However, the same authors presented a scenario for the 21st century in which the mean global temperature increases, and precipitation decreases, with warming felt more in summer and reduced precipitation occurring mainly in winter. The relationship between decreased precipitation and the subsequent increase in temperature is directly related to an increase in extreme drought phenomena, which in turn contributes to the occurrence of increasingly recurrent and severe fires.

#### 2.3.2. Fire as a Shaping Agent of the Mediterranean Landscape

Five regions of the world share a similar climate and structurally identical plant communities. These five regions, known as Mediterranean-type climate regions, as described by Keeley [30], are dominated by evergreen sclerophyllous shrublands, semideciduous scrub, and woodlands. These vegetation types are prone to widespread crown fires, and the common drought periods in summer represent an annual fire hazard that contributes to a highly predictable fire regime. Fire plays an important role in the development of these systems, as reflected in plant traits such as lignotubers in resprouting shrubs and delayed reproduction, which restrict recruitment to a pulse of post-fire seedings. These species, when installed in fertile soil, can resprout very quickly in the post-fire period, and opportunities for post-fire seedling recruitment are limited. Thus, these woody rates did not evolve in the way of delaying reproduction, as indicated by Keeley. In a review addressing the effect of fire on Mediterranean climate ecosystems, the author states that this fire-independent mobilization is common to the flora in the regions of the Mediterranean basin and in California, whereas post-fire seeding is more frequent in more arid regions due to their own geological specificities, with soils that are very poor in nutrients; therefore, the post-fire resprouts do not present such competitive behavior to seedlings, as post-fire seeding is more common. In Chile, also described by Keeley, there seems to have been fire-prone landscapes in the Andean uplift in the Tertiary, which were lost during the Miocene, which now prevent summer lightning storms from reaching the region.

**Figure 4:**
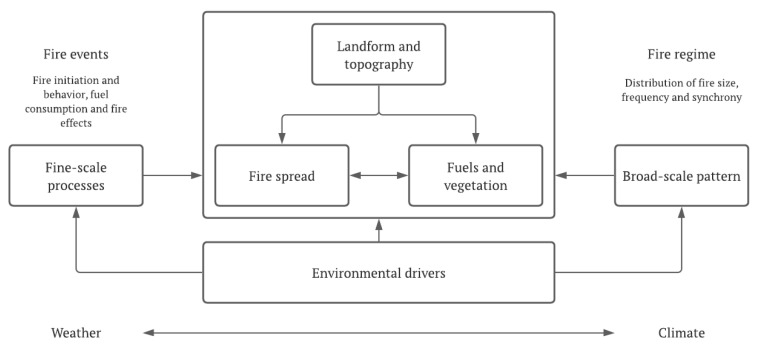
Cross-scale interactions of pattern and process in fire regimes. Environmental drivers of weather and climate, interacting with landform, set the overall template for individual fire events and fire regimes. Interactions between fire spread and vegetation determine the properties of fires at fine scales, while creating broad-scale landscape pattern [33].

Currently, all of these regions encounter serious problems with fire management, caused by the increased risk of fire, especially during the hottest and driest season, but also by the amount of highly flammable vegetation that accumulates during the remaining seasons of the year. It is also important to note that periodic fires are an important natural process in Mediterranean-climate ecosystems (Figure 4). However, as mentioned by Syphard et al. [31], the increased recurrence of fires profoundly threatens the fragile ecological systems of these regions. They also address the issue of the origin of fires, pointing to human action as the main cause, and relate population density with their occurrence. They conclude that there are few instances of human ignition in areas with low population density; therefore, fire frequency is low. On the other hand, when population density increases, human ignition and fire frequency also increase, at least until a certain threshold is reached. A similar relationship was obtained by Nunes et al. [32], who analyzed the evolution of fires between 1975 and 2020 in a region of northern Portugal. The authors related the occurrence and distribution of fires, verifying that increased recurrence of fires is directly related to increased population density, especially in a system of territorial organization with population nuclei being dispersed and interspersed with forestry and agricultural spaces. The authors also refer to human action as the main cause of the occurrence of fires, similar to what was reported by Syphard et al. for California (USA); therefore, it can be assumed that it is most likely a cause that extends to all Mediterranean-type climate regions.

#### 2.3.3. Social and Economic Impacts of Climate Change on Mediterranean Forests

**Figure 5:**
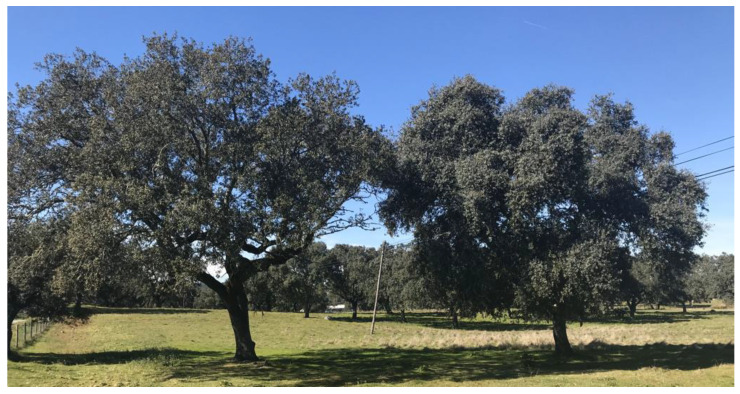
“Montado” landscape in Southern Portugal, in the region of Alentejo with a forest of *Quercus suber*.

The forests that grow in regions with a Mediterranean climate and fall within the group of temperate forests have always been the object of exploitation by human populations, who learned to use the resources made available by forests [34]. In the regions surrounding the Mediterranean basin, this interaction reached levels of near symbiosis, with populations interacting with forests in such a way that shaped both and created agro-forestry–pastoral systems, of which the “montado” in Portugal and the “dehesa” in Spain are examples [35,36]. In these areas, where the management activities of *Quercus suber* and *Quercus rotundifolia* forests are mixed with agricultural practices in dryland conditions, alongside the raising of animals (sheep, goats, cattle, and pigs), populations shaped the landscape over thousands of years, adapting it to their needs, but it also adapted itself to the available resources, which have always been managed sustainably [37]. This interaction between populations and the natural space (albeit partially artificialized) lead to the development of a culture that today identifies itself as Mediterranean, which, despite the differences that exist between the peoples of the Mediterranean basin, has commonalities to all, always related to the landscape that unifies them [38]. Figure 5 shows an example of the “montado” landscape.

After thousands of years of development of a culture supported by the territory from which it was born, in which traditional practices allowed populations to develop and prosper, profound changes in regional characteristics emerged and changed the way certain practices continue to develop [39]. In fact, all practices associated with the agro-forestry-pastoral environment are dependent on the succession of seasons and the climatic conditions of these regions, which in turn led to the development of specific types of floristic associations. However, with climate change, these conditions have been profoundly altered, leading to variations in the productive cycles of the species, with increased temperature in the summer and reduced precipitation in the winter, along with other problems, such as the arrival of invasive exotic species that directly compete with native species [40].

**Figure 6:**
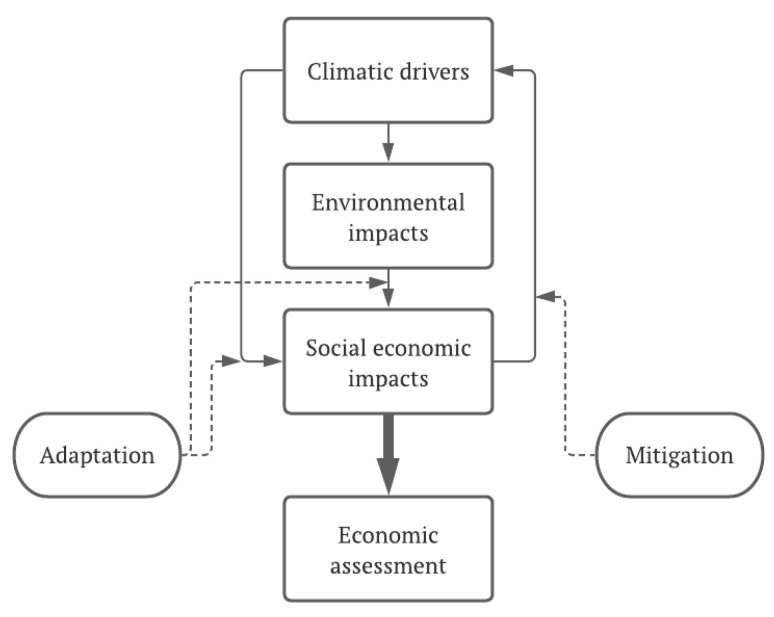
Socioeconomic impacts of climate change (adapted from https://www.iemed.org/publication/the-economic-impacts-of-climate-change-in-the-mediterranean/, accessed on 22 December 2021).

Fire, which has always been a part of Mediterranean ecosystems as an agent modelling the landscape and conditioning the evolution of the territory and the species that inhabit it, began to play a destructive role, contrary to what happened in the past when occurrences of fire spaced out in time contributed to the regeneration of crops and the elimination of pests and diseases [41]. Currently, with the changes in climatic parameters, fires have started to have a destructive role, with increased frequency and, mainly, in severity, with very significant impacts from an economic and social point of view [42]. The destruction of forest resources leads to the loss of a set of economic activities, including those of an industrial nature, such as cork production, but also traditional practices within the field of agriculture and pastoralism that also contribute to the income of rural populations [43]. This destruction of economic resources, in turn, leads to the emergence of problems and social disorder, through the elimination of jobs [44]. However, the social component is not just about the destruction of economic value, as the landscape component and people’s enjoyment of natural spaces are also part of the well-being component, whether from the perspective of enjoyment of the people who live in the vicinity or the perspective of enhancing the landscape, for example, for tourism purposes, as is presented in Figure 6 [45].

## 3. Forest Management in a Climate Change Scenario

Forest management in current times is forced to adapt to the new reality in a scenario of climate change, which implies major impacts and modifications in forest ecosystems. As previously mentioned in the work of Spittlehouse and Stewart [46], climate change is expected to have significant impacts on forest ecosystems over the next hundred years; therefore, surely practices associated with forest management must adjust to the new reality. It is increasingly expected that forest management will assume a position of contributing to the sustainability of resources, with an integrative vision that not only encompasses the traditional management of forest productivity as a supplier of raw materials, but also includes services that forests can provide, namely ecosystem services such as carbon capture and sequestration.

In the current scenario, which implies profound changes in climate parameters, with increased temperature and decreased precipitation, problems related to invasive species are added, which greatly contributes to uncontrolled growth and the accumulation of fuel load, which in turn significantly increases the risk of rural fires. In fact, as mentioned in the work by Seidl et al. [47], considering climatic uncertainties in management planning is a prerequisite for sustainable forest management, since it is also understandable that, as Linder [48] noted, projected climate change will strongly impact forest growth and composition, with obvious impacts on the exploration and enjoyment of forest resources. Spittlehouse [49], following this perspective, states that climatic change will affect society’s capacity to profit from forest resources.

Based on the scenario in which climate changes lead to an expected increase in temperature, maintaining the structure and functions of Mediterranean forests has become a challenge for forest managers. Vila-Cabrera et al. [37] showed that research is focused mainly on strategies to decrease risk and promote resistance in the short term, rather than on enhancing long-term resistance. On the other hand, as expected in economic activities, management strategies seek to obtain benefits in the short term and frequently have unintended consequences on other adaptation objectives and untargeted ecosystem components that are so important in Mediterranean-type climate regions.

## 4. Discussion

Forest management presents a set of problems that are now enhanced by the impacts of climate change, namely changes in climate parameters such as temperature and precipitation, and other underlying problems, such as an increased risk of fires, the spread of invasive species, and the emergence of pests and diseases [50]. Although of a different nature, these problems have some points in common, as they all contribute to the loss of productivity in forests, the degradation of the natural space, the loss of ecosystem services provided by forests, and the loss of biodiversity [51]. In addition to these negative consequences, they also have a common point, as they are all directly and indirectly related to the impacts caused by climate change [52]. In fact, these problems have always been described in the literature; therefore, it can be considered that they have always existed as part of forest ecosystems [53]. However, especially in the last two decades, all of these problems intensified to such an extent that it is practically impossible for them to go unnoticed.

Taking as an example the occurrence of fires, there is growing recurrence all over the globe, even in locations where fires occur infrequently; they were frequent in Mediterranean-type climate regions such as Portugal, Australia, and California (USA), but the same cannot be said of Sweden and Alaska (USA) [54]. In other words, climate parameters such as average air temperature and precipitation are changing in such a way that regions where fires were less likely to occur are now also experiencing more fires [55]. As has been widely reported in the media around the world, these fires, even in boreal forest regions, reached very significant dimensions, which were previously not common [56]. On the other hand, in temperate and tropical regions, where fires have always been frequent, they are now reaching truly catastrophic proportions, remaining active for weeks or months, making it impossible for fire-fighting forces to extinguish or control them with the means available, with increasingly high costs, as shown in Table 2.

However, the changes in temperature and precipitation are not only responsible for the intensification of and changes in patterns of occurrence of forest fires [57]. The changes in climatic parameters also interfere very significantly with the resilience of forest species, weakening them and making them more vulnerable to attacks by pests and diseases [58]. In this way, weakened forests will lose their competitive capacity, acquired during evolution over time in a given location and under certain edaphoclimatic conditions, due to the arrival of invasive species, many coming from regions with conditions identical to those that exist now [59]. This competition between native and exotic species usually leans on the side of newcomers, which are more aggressive, quickly occupying spaces in the ecosystem left empty by native species unable to adapt to new conditions [60]. In turn, these invasive species usually have the capacity to grow much faster than native species and are associated with a very large dispersal capacity, making them capable of accumulating large amounts of biomass that will also act as a fuel load to increase the risk of fires, creating a kind of vicious circle in which fire works as an agent of artificial selection and promotes and benefits pyrophyte and heliophile species [61]. In this way, the replacement of forest cover happens at the speed at which fires reach a certain region.

Forest management must assume the primary task of assessing the state of vulnerability of forests in the face of climate change as a starting point for the planning of management methodologies, but also include a review of expectations in terms of the use of forests, identifying needs at the level of research and education, developing forest policies to facilitate adaptation, and identifying the moment of implementation of responses, as described by Spittlehouse. Additionally, along this line of thought, Ogden and Innes [62] point out the need to articulate the specific objectives for adaptation to climate change, which the authors consider to be coincident with the criteria assumed for the conservation and sustainable management of forests. In a second stage, and because forest management plans are hierarchical (there are higher-level strategic plans and lower-level operational plans), it is important to distinguish which planning level is most appropriate to consider options.

**Figure 7:**
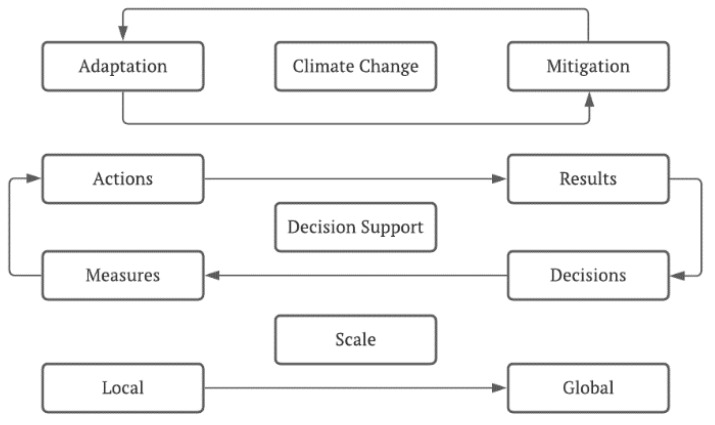
Integration concept of adaptive forest management [63].

On this assumption, and according to Bolte et al. [63], implementing an adaptive type of forest management can help forest ecosystems to adapt more easily to the new conditions so that the objectives of the management model are achieved while maintaining the ecosystem services and reducing the risk of forest degradation. As a management strategy, the authors presented (i) conservation of forest structures, (ii) active adaptation, and (iii) passive adaptation, with the criteria for applying each strategy depending on the intended management objective. For those authors, forest adaptation may entail the establishment of neonative forest, including the use and intermixing of native and non-native tree species, as well as trees with non-local provenance that may adapt better to future climate conditions, using an integrative concept of adaptive management that combines species suitability tests and modelling activities, priority mapping of adaptation strategies, and implementation. This approach to the concept of integrative adaptive forest management combines actions on different spatial scales based on their interaction regarding greenhouse gas mitigation and forest adaptation. In this way, actions that lead to forest adaptation at a local scale will have an influence on the success of global greenhouse gas mitigation, which will in turn contribute to triggering climate change and adaptation pressure at the local level, as shown schematically in Figure 7.

The existence of so many interrelations and interdependencies within a forest ecosystem with high biodiversity such as the Mediterranean requires a holistic approach that encompasses the entire ecosystem, and does not focus solely on forest species, but also involves other species from all biological kingdoms. As Bolte et al. reported, research and planning efforts must be intensified to meet all information needs regarding the ecosystem under consideration, so that the adaptation of the forest system can be included in the overall adaptive land-use management model.

Planted forests will also suffer from the impacts of climate change, and the adaptation of forest management to the new reality requires knowledge of the effects of climate change on forests, associated industries, and communities [64]. Keenan [65] states that the prediction of how effects might change over time and how to incorporate this knowledge in management decisions depends on new approaches to forest management. This author points out that the most relevant themes that should receive particular attention are (i) predicting species and ecosystem responses to the future climate, (ii) adapting actions for forest management, (iii) developing new approaches and tools for decision-making under uncertainty, and (iv) making policy arrangements for adaptation in forest management. This adaptive capacity assumes particular importance, especially when it is expected that there will be significant impacts on forest ecosystems over the next hundred years, as described by Spittlehouse and Stewart [46], who pointed out the need to evaluate the long-term effects on forests, and thus determine what measures should be implemented now and in the future to respond to the threat.

**Figure 8:**
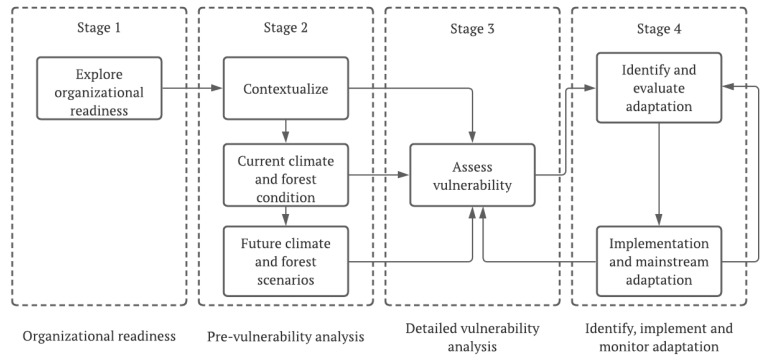
Four stages of adaptation to climate change in the context of sustainable forest management [66].

Thus, the foreseeable impacts are not limited to the loss of productivity of forest resources or of biodiversity, or even ecosystem services, but could also include the capacity of forests as an economic resource. Forests are sources of raw materials and support many activities that contribute to the creation of income and wealth; therefore, they have high social impact. However, the destruction of activities such as hunting and the collection of wild fruits, mushrooms, and nuts, including destruction of the landscape itself, with direct impacts on tourism, can put the very livelihoods of rural populations at risk. This can cause serious social consequences, with the flight of these populations to cities, further enhancing the already complex problem of the organization of territories and the asymmetries between rural and urban populations. Combating climate change is, therefore, an urgent need, as only by implementing mitigation measures, including measures related to sustainable forest management, will it be possible to mitigate, in a first phase, and then reverse the catastrophic effects of radical climate change in the following stages, as presented in Figure 8.

## 5. Conclusions

Climate change is a problem that affects everyone on a global scale, with a profound impact on ecosystems. Forest ecosystems are susceptible to climate change, and are affected in different ways, ranging from the loss of forest productivity to the loss of the ecosystem services they provide, such as the capture and sequestration of atmospheric carbon. Although all forests are affected by climate change, those in Mediterranean-type climate regions have greater susceptibility, derived from the effects of climate change on parameters such as temperature and precipitation. These parameters affect the development of forest species by weakening them, which potentiates the emergence of diseases and pests and invasive species, and an increased risk that even more intense and severe fires will occur. In this way, the implementation of adaptive forest management systems is presented as a need so that forests can become more resilient and able to adapt to the new reality, and, thus, also to contribute to climate change mitigation.

## Figures and Tables

**Table 1:** Proportion of forest area by climatic domain in the year 2020 (adapted from https://www.cepf-eu.org/news/two-important-reports-published-worlds-forests, accessed on 22 December 2021).

Forest Type	Proportion (%)
Tropical	42
Boreal	27
Temperate	16
Subtropical	11

**Table 2:** Average annual fire suppression expenditure for the emergency fund of California Department of Forestry and Fire Protection (adapted from https://www.statista.com/chart/19807/california-wildfire-emergency-fund-expenditure, accessed on 22 December 2021).

Decade	Cost (USD)
1979–1989	25,000,000
1990–1999	61,000,000
2000–2009	236,000,000
2010–2019	401,000,000

## Data Availability

The data presented in this study are available by request from the corresponding author.
